# Effects of assisted reproductive technology on gene expression in heart and spleen tissues of adult offspring mouse

**DOI:** 10.3389/fendo.2023.1035161

**Published:** 2023-03-30

**Authors:** Huanhuan Chen, Lei Zhang, Feng Yue, Chenchen Cui, Yan Li, Qingwen Zhang, Linlin Liang, Li Meng, Cuilian Zhang

**Affiliations:** ^1^ Reproductive Medicine Center, Henan Provincial People’s Hospital, People’s Hospital of Zhengzhou University, Henan Provincial People’s Hospital of Henan University, Zhengzhou, Henan, China; ^2^ Henan Joint International Research Laboratory of Reproductive Bioengineering, Zhengzhou, Henan, China

**Keywords:** assisted reproduction, gene expression, heart, spleen, offspring, mouse

## Abstract

**Objectives:**

Assisted reproductive technology (ART) is an important part of reproductive medicine, whose possible effects on offspring’s health have drawn widespread attention in recent years. However, relevant studies are limited to postnatal short-term follow-up and lack of diverse sample sources analysis other than blood.

**Methods:**

In this study, a mouse model was used to explore the effects of ART on fetal development and gene expression in the organs of offspring in the adulthood using next-generation sequencing. The sequencing results were then analyzed.

**Results:**

The results showed that it caused abnormal expression in 1060 genes and 179 genes in the heart and spleen, respectively. Differentially expressed genes (DEGs) in the heart are mainly enriched in RNA synthesis and processing, and the cardiovascular system development also shows enrichment. STRING analysis identified *Ccl2, Ptgs2, Rock1, Mapk14, Agt*, and *Wnt5a* as the core interacting factors. DEGs in the spleen are significantly enriched in anti-infection and immune responses, which include the core factors *Fos, Jun* and *Il1r2*. Further exploration revealed the abnormal expression of 42 and 5 epigenetic modifiers in the heart and spleen, respectively. The expression of the imprinted genes *Dhcr7, Igf2, Mest* and *Smoc1* decreased in the hearts of ART offspring, and the DNA methylation levels of *Igf2-* and *Mest*-imprinting control regions (ICRs) increased abnormally.

**Conclusion:**

In the mouse model, ART can interfere with the gene expression pattern in the heart and spleen of the adult offspring and that these changes are related to the aberrant expression of epigenetic regulators.

## 1 Introduction

Assisted reproductive technology (ART) is an important branch of reproductive medicine that has developed rapidly in recent years. It comprises ovarian stimulation, oocyte and sperm collection, cryopreservation, *in vitro* fertilization (IVF), intracytoplasmic sperm injection (ICSI), *in vitro* embryo culture, embryo transfer and trophoblast or blastocyst biopsy. ART also has important applications in blocking genetic diseases and preserving the fertility of young tumor patients (1, 2). However, ART is utilized during a critical period of gamete and embryo development at a time when the genome is undergoing significant epigenetic remodeling, and changes in the environment can easily affect normal developmental programming (3).

ART is associated with low birth weight (4, 5). A recent study reported that children conceived by ART grew more rapidly after birth, gaining more height and weight in early childhood, but no significant difference was observed between these children and those who born naturally once they reached adulthood (6). In IVF, *in vitro* embryo culture and embryo transfer affect the growth and organ development of postnatal mice, and disturb metabolic homeostasis by causing systemic oxidative stress and mitochondrial dysfunction (7, 8). ART increases the risk of type 2 diabetes, metabolic syndrome and cardiovascular diseases in both human and mouse (9). The effect of ART procedures on the postnatal health of offspring is consistent with the developmental origin of health and disease (DOHaD) theory. The theory holds that the formation of lifestyle-related diseases in adults such as cardiovascular disease, diabetes, and chronic kidney disease, may originate from the fertilized oocytes, early embryos, fetuses and newborns as a result of the interaction between genes and the environment (10).

Recent studies have shown that ART increases the risk of cardiovascular disease in human and animal offspring and disturbs cardiac metabolism (3). ART is associated with cardiovascular remodeling *in utero* that persists postnatally (11). Compared with naturally conceived babies, ART newborns have a higher risk of developing congenital heart disease (CHD) (12), and the blood pressure of the offspring is also likely to be higher (13–15). It has been found that ART offspring are prone to poor cardiac diastolic function and thickening of the vascular wall (16). Similar phenomenons were observed in the ART mouse model. Vascular dysfunction and arterial hypertension occurred in mice generated by ART (17). Mouse embryo culture and transfer led to elevation of systolic blood pressure at 21-week-old offspring mice, and the increase in the activity of cardiovascular metabolism-related enzymes significantly in 27-week-old female offspring (18). The mouse system can, consequently, be suitably used to study, modify, and improve the outcomes for ART children (9). In addition, the application of ART affects the immune status of newborns, for example, high E2 levels before embryo transfer increase IL-4 levels (19). ART offspring mice also have reduced immunity, with the significantly decreased intracellular expression of T-bet and serum levels of IFN-γ and a marked increase in IL-4 and IL-17A (20). However, the effect of ART on gene expression in the hearts and spleens of offspring and its mechanism are unknown.

The health and developmental problems of ART offspring may be closely related to epigenetic changes (21). There is evidence that ART increases the risk of epigenetic disorders in humans and animals, which lead to adverse outcomes later in life (3, 22). Multi-omics studies revealed that ART mainly influences DNA methylation and the histone modification of H3K4me3 in human offspring (23). There are also extensive epigenetic changes in children with a normal ART phenotype. DNA methylation changes occur not only at the whole genome level, but also in multiple imprinted genes, increasing the risk of cardiac metabolic abnormalities (24). There are specific DNA methylation patterns in ART neonates, including imprinted and housekeeping genes, involving neural and immune system pathways (25). Studies have shown that ovulation induction results in imprinting errors and cross-generational effects (26).

According to the current research findings, ART has the potential for causing adverse effects on the growth, development, and metabolism of human and animal offspring, including an increased risk of cardiovascular disease and abnormal immune function. We believe that phenotypic abnormalities and the potential risks caused by ART may be related to changes in gene expression in the corresponding organs, on which there is no relevant research at present. Therefore, using a mouse model and high-throughput sequencing, this study analyzed the effect of a series of ART procedures on gene expression in the heart and spleen of adult offspring, thereby revealing the significantly affected pathways and core genes, including epigenetic modification-related and imprinted genes.

## 2 Materials and methods

All chemicals and reagents were purchased from Sigma-Aldrich (St. Louis, USA), except those specifically indicated otherwise.

### 2.1 Feeding, management and use of mice

The CD-1 (ICR) mice were kept and used following the guidelines for the care and use of laboratory animals of Zhengzhou University, Henan, China. They were raised in a 12/12 h light/dark cycle at 25°C and 50% humidity. All animals were placed in independently ventilated cages, where they could eat and drink ad libitum. Every three same-sex animals were placed in a cage. The offspring were raised to adulthood at 8 weeks. The experimental mice were sacrificed by cervical dislocation. The breeding, management, and use of the mice followed the “3Rs” principle of animal ethics.

### 2.2 Ovulation induction, vitrification and thawing

A total of 10 eight-week-old female mice were selected for ovulation induction. Each mouse was injected with 10 IU of pregnant mare serum gonadotropin (PMSG, NSHF, China) and 10 IU of human chorionic gonadotropin (hCG, NSHF, China) after 46 h. Cumulus oocyte complexes (COCs) were obtained from the ampulla of the oviduct after 15 h, and the cumulus cells were removed by hyaluronidase digestion. The gamete buffer was M2 medium (5.42726 g/L HEPES, 0.35 g/L NaHCO3, 1.0 g/L glucose, 0.0106 g/L phenol red, 0.0363 g/L sodium pyruvate). According to the release of the first polar body, MII oocytes were selected for cryopreservation. The Cryotop method was used for oocyte vitrification. The main procedures of vitrification and thawing were the same as our previous report (27). The solutions include equilibrium solution (ES): 7.5% (v/v) ethyl glycol (EG), and 7.5% (v/v) dimethyl sulfide (DMSO) and the vitrification solution (VS): 15% EG, 15% DMSO, 30 g/L Ficoll‐70, and 0.5 M sucrose. The base fluid was a modified phosphate buffer solution (PBS) containing 3mg/ml BSA (0.2 g/L KCl, 0.2 g/L KH2PO4, 8 g/L NaCl, 1.15 g/L Na2HPO4, 3mg/ml BSA). Briefly, the oocytes were first soaked in the ES for 5–12 min and then transferred to the VS after the morphology was completely restored. Within 1 min, the oocytes wrapped with a small amount of liquid were transferred to the Cryotop straw and then quickly inserted into liquid nitrogen to complete the vitrification. After one year, the frozen oocytes were thawed. According to the order of use, the thawing solution includes thawing solution (TS), diluting solution (DS), and washing solution (WS), containing 1.0, 0.5, and 0 M sucrose in the base fluid, respectively. The oocyte soaking time in the thawing solution was 1, 3, and 5 min, respectively. The use temperature of the TS was 37°C, and the rest were room temperature. After thawing, the oocytes were cultured in potassium simple optimized medium (KSOM, IVL04-100ML, Caisson labs, USA; medium composition provided in **Supplementary Materials**, **Table S1**) for 1 h at 37°C with 5% CO2 before fertilization to promote the recovery of the cytoskeleton.

### 2.3 *In vitro* fertilization, embryo culture and transfer

Fresh mouse spermatozoa were obtained from the cauda epididymidis and the vas deferens of three adult male mice that had successfully mated. After the epididymal tail and vas deferens were cleaned with G-IVF™ plus (10136, Vitrolife, Sweden; medium composition provided in **Supplementary Materials**, **Table S1**), they were cut with ophthalmic scissors and then gently pressed to make the sperm overflow. The sperm was transferred to the bottom of the pre-balanced 15mL centrifuge tube containing G-IVF™ plus and then captured for 40 min at 37°C with 5% CO2 using the upstream method. The supernatant of the sperm was taken and placed in a new centrifuge tube. After centrifugation, the capacitated sperm was obtained. It was washed once with G-IVF™ plus, and a few drops of sperm were smeared on the slide to observe the morphology and vitality. If most of the sperm moved in a straight line and had a complete tail, it was judged as qualified. A small amount of sperm was taken and incubated at 50°C for 5min, and then a blood cell counting board was used to count the sperm. To improve the low fertilization rate of the vitrified oocytes, a Pizol oscillator (Primetech, Ramat‐Gan, Israel) connected to a micromanipulator was used to “punch holes” in the zona pellucida: each oocyte had three holes at different positions. The treated sperm capable of fertilization and the oocytes were incubated in 50 μL droplets of G-IVF™ plus under mineral oil at 37°C with 5% CO2. Each drop contained about 40 oocytes and the final concentration of sperm was 2.0 × 10^6^/mL. After 5 h, 2PN zygotes were selected and washed under a stereomicroscope before the embryo culture. After being cultured for 24 h in KSOM at 37°C with 5% CO2, the embryos developed into the two-cell stage, and 25 embryos were surgically transplanted into the ampulla of the oviduct of each pseudopregnant female. These mice were obtained by mating naturally estrous female mice with male mice with vasoligation and a total of seven successfully mated. During the embryo transfer, the recipient female mice were under general anesthesia and placed in a prone position. An incision was made at the corresponding position of the ovary on the back side under sterile conditions to expose the ovary and oviduct and fix them. The ampulla of the oviduct was found through the anatomy microscope. A pair of microscissors was used to cut a small opening, and then the embryo transfer tube (self-made capillary glass tube) was inserted. After entering a certain distance towards the uterus, the pre-drawn embryos were injected. By the time the transfer finished, the muscles and skin were sutured and disinfected. The animals were subsequently kept in the vivarium until the studies were complete. From birth to 8 weeks old, the weight of the offspring was recorded.

### 2.4 Acquisition of tissue samples

For the sequencing research, three individual samples that were obtained from different litters were included in each group. After the offspring female mice at 8 weeks old were killed by cervical dislocation, half of the heart tissue from the apical area (equivalent to the ventricular part) and the whole spleen were taken. The heart and spleen tissues were washed with normal saline to remove the blood. Then, they were cut into 0.5 × 0.5 cm pieces using sterile RNase-free surgical scissors. Liquid nitrogen was added so that the samples could be ground into powder, after which they were placed into a sterile RNase-free EP tube, and TRIzol solution was then added to fully lyse them. Later, the total RNA for RNA sequencing (RNA-seq) according to the conventional RNA extraction steps was obtained.

### 2.5 Transcriptome sequencing and bioinformatics analysis

First, Agilent 2100 was used to test the concentration, purity and RNA integrity number (RIN) of the prepared RNA samples. For the qualified samples, the standard Illumina protocol was carried out to construct cDNA libraries comprising ribosome RNA (rRNA) removal, messenger RNA (mRNA) isolation and fragmentation, complementary DNA (cDNA) double-strand synthesis and purification, cDNA terminal repair, 3’ end plus A, adaptor ligation, screening cDNA fragments of around 200 bp, PCR library enrichment and purification. Then, sequencing was performed through the Illumina hiseq2500 platform. For the raw sequencing reads, the adapter sequence and low-quality bases were removed through Fastp (version 0.18.0) to obtain clean reads. After filtering out the rRNA reads, genome mapping was performed on the clean reads using Hisat2 (version 2.4). Then, gene expression was calculated by STRINGtie (version 1.3.1), and the results were expressed by fragment per kilobase of transcript per million mapped (FPKM). To screen out the differential genes between the groups, R package DESeq2 suitable for small sample size was used with stricter cutoff values. The screening criteria for differential genes were the following: the FPKM in at least one group was greater than 1; the differential fold change (FC) was greater than 2.5; and the false discovery rate (FDR) was less than 0.05. For differentially expressed genes (DEGs) between the groups, GO and KEGG enrichment analyses were performed. Using the STRING online tool, the protein interaction of the screened DEGs was analyzed. The general steps included selecting “multiple proteins”, inputting the gene list to be analyzed, generating a protein interaction network according to the default parameters, and then performing K-means clustering with the default number of clusters.

### 2.6 qPCR validation of differential genes

Through a real-time fluorescent quantitative PCR (qPCR) test, the expression of certain differentially expressed genes (DEGs) found in sequencing analysis was verified, including functional, imprinted, and epigenetic modification genes. First, the total RNA extracted using the TRIzol method was reverse transcribed (AT311, TransGen Biotech, China) to obtain cDNA. The Primer-BLAST tool fromNCBI was used to design specific quantitative detection primers (**Table S2**). The quantitative PCR enzyme was TB green^®^ Premix Ex Taq ™ II (RR820, Takara, Japan), the detection instrument was a Roche LightCycler^®^ 96, and the PCR reaction conditions were 95°C for 50 s, 95°C for 5 s, and 58°C for 30 s at 40 cycles. Each PCR reaction contained 50ng of template cDNA. Based on the Ct values of the target and the reference genes, the relative expression difference between the groups was calculated by 2^−ΔΔCt^. The internal reference gene *GAPDH* was chosen. The PCR amplification efficiencies for the primers of the reference gene and the selected DEGs were verified. Each group consisted of five biological replicates, which were from different litters.

### 2.7 DNA methylation analysis of imprinted genes

For the imprinted genes in the offspring tissues affected by ART, bisulfite sequencing PCR (BSP) was further used to detect DNA methylation changes in the imprinting control region (ICR). After genomic DNA is treated with bisulfite, cytosine (C) at the unmethylated CpG dinucleotide is converted to uracil (U), which corresponds to thymine (T) after PCR amplification, while the methylated CpG remains unchanged. First, the genomic DNA was extracted from the control and experimental group tissues. Then, the DNA was subjected to C–T conversion using an EZ DNA Methylation-Direct™ Kit (E2003, Zymo Research, USA). Finally, using the transformed DNA as a template, a sequence on the ICR was amplified by nested PCR. The BSP primer sequences for Igf2 (28, 29) and Mest (30) were taken from the literature (**Table S3**). The PCR amplified product was analyzed using Sanger sequencing and compared with the original sequence to identify the DNA methylation sites. The ICR of the imprinted gene Mest is located in the 5’ region-harboring presumptive promoter (GeneBank acc. no. AF017994) (31). The ICR region of H19/IGF2 is also confirmed to be located in the 5’ non-transcribed region (GeneBank acc. no.U19619) (32, 33). The amplified Mest and Igf2 products contained 23 and 15 CpG sites, respectively. Each group consisted of five biological replicates, which were from different litters. An independent sample Student’s *t* test was used to analyze the statistical difference of the methylation level between the groups, and the difference was significant if *p* < 0.05.

### 2.8 Experimental design

To simulate a realistic procedure that a woman would undergo, the treatment group underwent a series of ART procedures including superovulation, egg freezing, IVF, and embryo culture and transfer. In the ART group, the female mice were ovulated with PMSG and hCG, and the MII oocytes were vitrified. After thawing, IVF was carried out. The embryos were cultured *in vitro* to the two-cell stage and then surgically transplanted. The female offspring were cultured to adulthood. In the control group, female mice in natural estrus were selected to be caged with adult male mice. After successful natural mating, the offspring mice were raised to 8 weeks. Heart and spleen tissues of the female offspring from the two groups were taken, and the total RNA was extracted for transcriptome sequencing and qPCR validation.

## 3 Results

### 3.1 ART offspring birth and growth, transcriptome sequencing and sample relationship analysis

**Figure 1:**
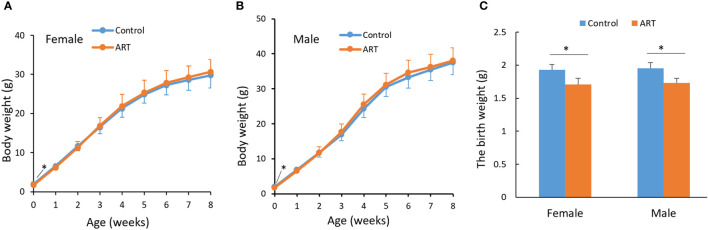
Growth curve of mice within eight weeks after birth. **(A)** Body weight of female offspring; **(B)** body weight of male offspring; **(C)** a histogram showing the birth weight of the offspring mice. Blue and orange lines represent the control group and ART group respectively. N equals 10 in all groups. Values are means ± SD. “*” represents a statistically significant difference by t-test (p < 0.05).

We counted the litter sizes of the control group and the ART group and recorded the weight of the offspring mice from birth to 8 weeks. As a result, the fertilization rate of oocytes in the ART group was 46.3%, the two-cell rate was 87.5%, and the birth rate of the ART offspring was 39.2%, relative to the number of two-cell embryos transferred (**Table 1**). Both the control group and the ART group had 5 litters of offspring mice, of which the former contained a total of 51 progenies (10, 9, 6, 12, 14), while the latter was made up of 49 progenies in total (13, 10, 7, 11, 8). No significant difference was observed in the litter size between the two groups, while the average body weight of the newborn mice in the ART group was significantly lower than that in the control group. It was worth noting that the difference in the body weight between the two groups disappeared during the subsequent growth (**Figure 1**).

**Table 1:** Effects of ART on preimplantation embryo development and birth of offspring mice.

Groups	Fertilization rate (%)	two-cell rate (%)	Litters	Total offspring	Litter size
Control	NA	NA	5	51	10.20 ± 3.03 (NA)
ART	46.3 ± 2.3	87.5 ± 1.7	5	49	9.80 ± 2.39 (39.2%)

The fertilization rate refers to the ratio of 2PN zygotes to the oocytes survived after vitrification. The two-cell rate was calculated relative to the number of fertilized oocytes. Values are shown as mean ± standard deviation. “%” refers to the ratio of the total number of ART offspring to the total number of transferred two-cell embryos.

NA, Not Applicable.

**Figure 2:**
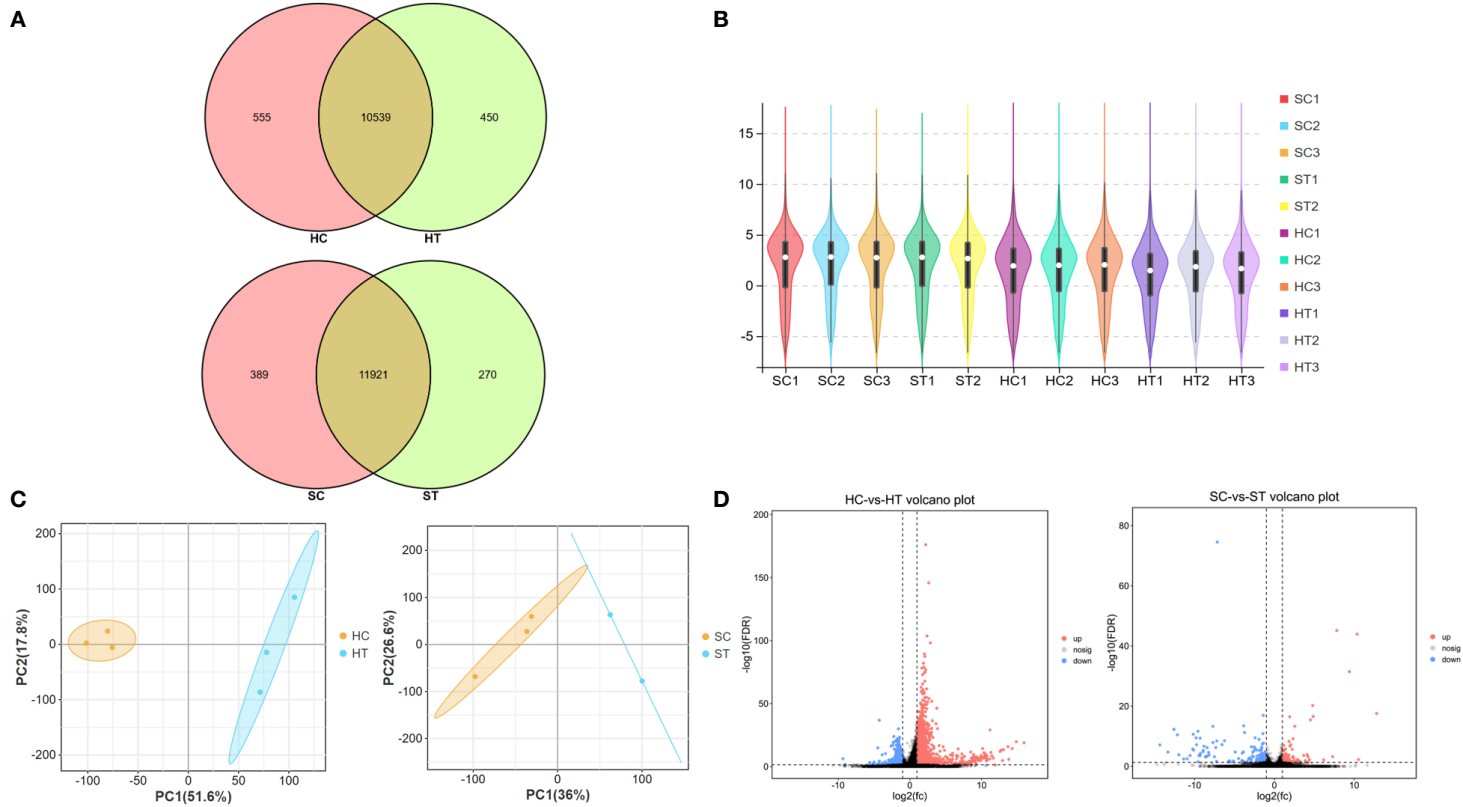
Assisted reproductive technology **(**ART)**-**changed gene expression profiles in heart and spleen tissues of offspring. **(A)** Venn diagram shows the number of genes that are co-expressed and specifically expressed between groups; **(B)** violin plot shows the distribution of gene expression in the sequenced samples; **(C)** principal content analysis (PCA) result of heart and spleen samples, respectively; **(D)** volcano plot indicates the differently expressed genes (DEGs) between ART and control groups in heart and spleen, respectively. HC and HT represent the heart tissue of the offspring of the control and ART group, respectively; SC and ST represent the spleen tissue of the offspring of the control group and the ART group, respectively.

To further explore the effects of ART on gene expression in adult offspring, the heart and spleen tissues of the control and ART adult female offspring were obtained for transcriptome sequencing. The amount of sequencing clean data was about 6 GB, and the base quality value Q20 > 97%, Q30 > 93% (**Table S4**). The number of raw reads per sample was 4–5 × 10^7^, and the proportion of clean reads was more than 96%. The clean reads were mapped to the mouse reference genome (Ensembl_release100), and the results showed that the mapped rates of the samples were more than 95%, and the unique mapped reads rate was 73–88%. The sequencing saturation of all samples was good, and the gene coverage detected in the heart was about 70% and close to 74% in the spleen (**Table S5**). Most genes were shown to be commonly expressed in the heart and spleen of the control and ART groups based on the venn diagram generated using the gene expression data. However, there were still some genes with differential expression between the two groups in the same organ. (**Figure 2A**). To show the distribution of gene expression in different samples and to demonstrate the quality of the database construction and sequencing, we further created a violin plot using the FKPM value of the gene expression (**Figure 2B**). Then, principal component analysis (PCA) was performed on heart and spleen samples separately, which illustrated the good repeatability of the samples within the groups (**Figure 2C**).

### 3.2 Effect of ART on the whole gene expression in heart and spleen tissues of adult offspring

**Figure 3:**
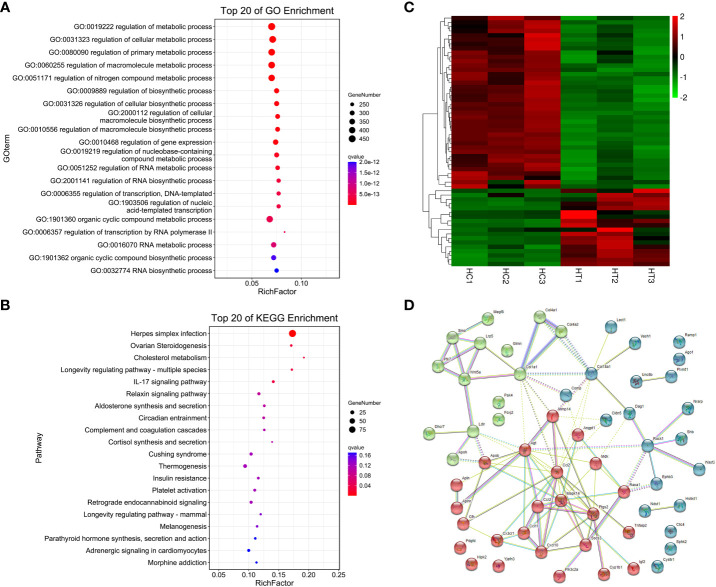
Functional enrichment and interaction analysis of DEGs in heart tissue of ART offspring. **(A, B)** GO and KEGG enrichment analysis of DEGs in the heart (top 20), respectively; **(C)** heatmap shows the 58 DEGs included in the term “cardiovascular system development”; **(D)** the interaction of the above 58 DEGs was analyzed by STRING software, and kmeans clustering was performed.

A total of 1060 genes were differentially expressed in the heart of the ART offspring, of which 497 were downregulated and 563 were upregulated (**Figure 2D**). The DEGs in the heart were then subjected to gene ontology (GO) and *Kyoto Encyclopedia of Genes and Genomes* (KEGG) enrichment analysis (**Figures 3A, B**; **Supplementary Materials**, **Table S6**). Five pathways were found to be significanly enriched for these DEGs, which were not related to cardiac function. Although cardiac muscle contraction did not show significant enrichment (*q* > 0.05), it contained five differential genes: *mt-Co2, mt-Cytb, Trdn, Myl4* and *Atp2a3*. The results of the GO enrichment showed that the most significantly enriched biological processes of cardiac DEGs were related to RNA transcriptional synthesis. The cardiovascular-related terms “circulatory system development” and “cardiovascular system development” were also revealed to be significant functional terms although they were not included in the top-20 terms. The former contained 82 differential genes (**Supplementary Materials**, **Table S6**), and the latter contained 58 (**Figure 3C**). The interaction and clustering analysis of these 58 genes were performed using the STRING online tool. The core genes included *Ccl2, Ptgs2, Rock1, Mapk14, Agt*, and *Wnt5a* (**Figure 3D**). qPCR verified the differential expression of *Rock1* and *Mapk14* (**Table 2**).

**Table 2:** qPCR validation of gene expression in offspring affected by ART.

Organ	Gene Symbol	Fold Change (RNA-Seq)	Fold Change (qPCR)
Heart	*IGF2*	0.38	0.27
	*MEST*	0.4	0.33
	*KDM2A*	0.4	0.31
	*MAPK14*	0.34	0.38
	*ROCK1*	3.17	4.25
*Spleen*	*KDM5D*	227.33	85.44
	*FOS*	27.85	18.36
	*JUN*	4.18	4.72

“Fold Change” indicates the ratio of gene expression in the vitrified group relative to that in the control group.

**Figure 4:**
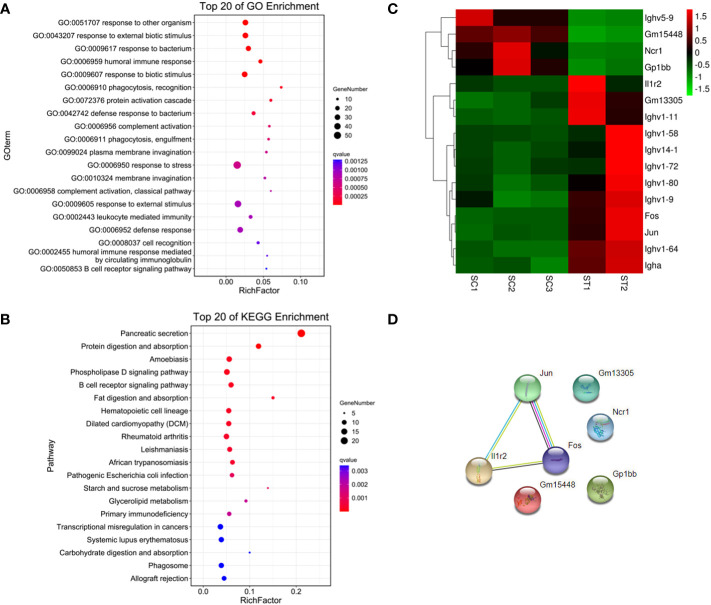
Functional enrichment and interaction analysis of DEGs in spleen tissue of ART offspring. **(A, B)** Gene ontology (GO) and *Kyoto Encyclopedia of Genes and Genomes* (KEGG) enrichment analysis of DEGs in spleen (top 20), respectively; **(C)** heatmap shows 16 DEGs included in immune related pathways; **(D)** the interaction analysis of the above 16 DEGs was performed using STRING software.

179 genes were found to have abnormal expression in the spleen, including 116 down-regulated and 63 up-regulated genes (**Figure 2D**). GO analysis found that these DEGs were enriched in some biological processes related to the immune response such as “defense response to bacteria”, “complex activation”, “phagocytosis, recognition” and “human immune response” (**Figure 4A**; **Supplementary Materials**, **Table S6**). KEGG analysis also displayed that these DEGs were significantly enriched in the immune-related pathways, including the “B cell receptor signaling pathway”, “hematopoietic cell lineage”, “primary immunity”, “natural killer cell mediated cytotoxicity”, and “Fc gamma R-mediated phagocytosis” (**Figure 4B**; **Supplementary Materials**, **Table S6**), in which 16 DEGs were enriched (**Figure 4C**). STRING analysis showed that *Fos, Jun* and *Il1r2* interacted with each other (**Figure 4D**). The differential expression of *Fos* and *Jun* was verified by qPCR (**Table 2**).

### 3.3 Effect of ART on expression of epigenetic modification-related factors in offspring tissues

**Figure 5:**
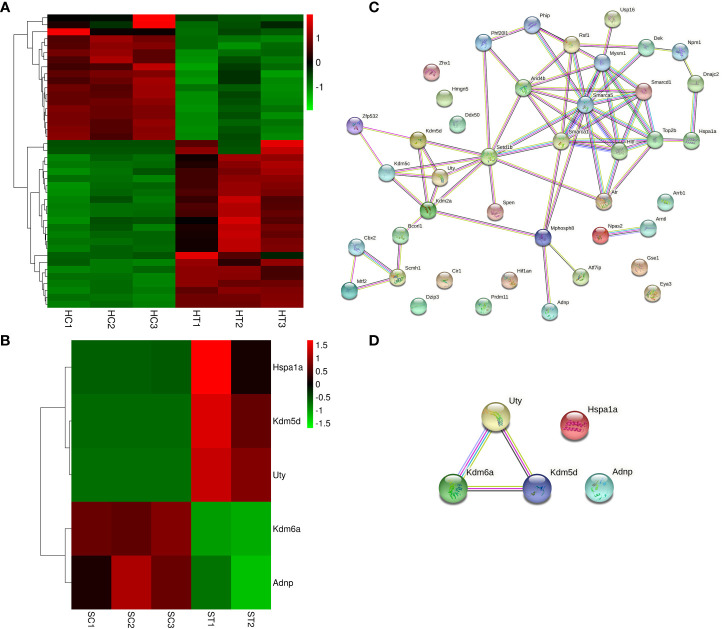
ART affects the expression of epigenetic modifiers in the heart and spleen of offspring. **(A, B)** Heatmap showing the effect of ART on the expression of epigenetic modifiers in the heart and spleen tissues of the offspring, respectively; **(C, D)** STRING analysis revealed the interaction between aberrantly expressed epigenetic modifiers in heart and spleen tissues of ART offspring, respectively.

The DEGs in the heart and spleen of ART offspring were compared with known epigenetic modifiers (https://epifactors.autosome.org/). The results showed that 42 genes were abnormally expressed in the heart (**Figure 5A**), among which *Ddx50* was related to RNA modification, *Mtf2* and *Scmh1* were Polycomb-group (PCG) proteins, *Npm1* is a histone chaperone, and the others were related to chromatin remodeling or histone modification. Five genes were abnormally expressed in the spleen (**Figure 5B**), of which *Adnp* was a chromatin remodeling cofactor, and the others were related to histone modification. To analyze the interaction between different epigenetic modifiers using STRING database, it was found that the core genes *Smarca5, Arid4b, Setd1b* and *Kdm2a* have interactions in the heart (**Figure 5C**), and *Kdm6a, Kdm5d* and *Uty* interacted in the spleen (**Figure 5D**). All of the above factors were related to histone modification or chromatin remodeling. The differential expression of *Kdm2a* and *Kdm5d* was verified by qPCR (**Table 2**).

### 3.4 Effect of ART on imprinted gene expression and methylation in offspring tissues

**Figure 6:**
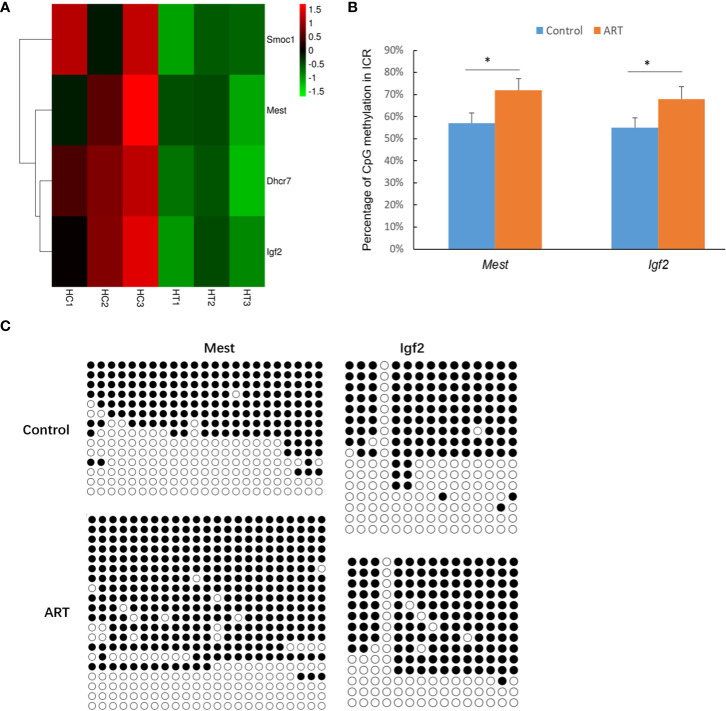
ART affects the expression and methylation of several imprinted genes in offspring hearts. **(A)** Imprinted genes with abnormal expression in heart tissue of ART offspring; **(B)** DNA methylation levels in imprinting control regions (ICRs) were detected by bisulfite sequencing PCR (BSP) method; **(C)** representative results of methylation of imprinted genes *Mest* and *Igf2*. Methylated and unmethylated CpG dinucleotides are represented by filled and open circles, respectively. Superscript “*” indicates significant difference between groups (*p* < 0.05).

DEGs in the heart and spleen of ART offspring were compared with known mouse imprinted genes in the public database (https://www.geneimprint.com/site/home). The results showed that the expression of four imprinted genes in the hearts of the ART offspring significantly decreased (**Figure 6A**). Among these, *Dhcr7* is a paternal imprinted gene, and *Igf2, Mest*, and *Smoc1* are maternal imprinted genes. There was no significant differential expression in the expression of imprinted genes in the spleen between control and ART groups. qPCR experiments verified the abnormal expression of *Mest* and *Igf2* (**Table 2**). Then, the effect of ART on the DNA methylation of *Mest* and *Igf2* ICRs was further investigated using the bisulfite sequencing PCR (BSP) method. The results showed that the DNA methylation levels of both ICRs significantly increased: *Mest*, 72 vs. 57%; *Igf2*, 68 vs. 55% (**Figure 6B, C**).

## 4 Discussion

ART is of great significance to treat conventional infertility couples, blocking the transmission of genetic diseases to offspring, preserve the fertility of patients with malignant tumors, and building human sperm and oocyte banks. Most of the children who were conceived using ART are no different in most respects from the children born through natural pregnancy. However, ART procedures occur at the critical period of gamete and embryo development, which may increase the health risks of the offspring after reaching the adulthood, especially concerning cardiovascular problems (3). Therefore, exploring the effect of ART on gene expression in the organs of the offspring of mice is helpful for answering this question.

Studies on human and animal models have shown that the impact of ART on offspring is often more significant after adulthood. Animal studies not only have confirmed the impact of human ART on the offspring cardiovascular system, immune system, adipose tissue, and liver, but can also be useful for observing the whole life cycle (3). In this study, animal models were used to explore how ART affected the growth of the offspring and gene expression profiles of their heart and spleen. In current clinical practice, human embryos are transferred at the blastocyst stage or sometimes at the cleavage stage. Zygotic genome activation (ZGA), an important molecular event in the preimplantation embryo development, occurs at different times among different species The ZGA in mouse occurs in the two-cell embryos, while that occurs at the four- and eight-cell stages in human (34). The conclusion of this mouse study was obtained by transferring the two-cell embryos. In humans, transfer of the six-to-eight-cell embryos instead of the two-cell embryo could result in different outcomes and hence future studies are needed to answer this question. By controlling the amount of embryos transferred, the number of litters in the ART group was similar to that in the control group, eliminating any possible interference caused by different litter sizes (16). We found that ART reduced the birth weight of the offspring mice, although the weight of the offspring after adulthood was normal. Previous studies have also reported that ART affects placental development and function, leading to fetal weight loss (35, 36). Embryo culture reduced the weaning weight of the offspring mice, although this difference disappeared in adulthood. The weight of the thyroid gland was reduced, while the weight of the spleen, heart and other organs were not significantly changed (7, 18). Less optimal embryo culture medium (Whitten medium) altered the weight and heart structure of adult male offspring mice, but optimal medium (KSOM) had no significant effect (37). However, a recent study showed that ART procedures such as ovulation induction, embryo culture and embryo transfer significantly increased the weight of offspring mice, resulting in a reduction in multiple organs/weight ratios such as the heart and spleen in 27-week-old female offspring (38). The PGD technology widely used in recent years was found to increase the weight of male offspring mice after two weeks of birth (39). The effect of ART on offspring weight may be related to abnormal glucose and lipid metabolism. IVF mouse offspring had an altered liver and serum metabolomic profile, which was mainly related to perturbed glucose metabolism (40). In addition, ART affected the fatty acid metabolism of male offspring mouse liver and adipose tissue, and this effect varies with the age of the offspring (41).

IVF-produced female offspring showed the altered expression of the renin–angiotensin system in the myocardium, which regulates the blood pressure and is known to play a role in the pathogenesis of cardiovascular disease (42). ART led to higher blood pressure and increased body weight in adult female offspring (3). The optimized ART procedures can still significantly affect the growth and metabolism of female offspring, including body weight, bone mineral density, fat content, fasting blood glucose, and insulin secretion (8). Therefore, female adult offspring were selected as study subjects for sequencing analysis in this study. According to previous studies, ART has a significant impact on the ventricular function in animals and humans (11, 37). In order to minimize the interference of other cardiac tissues on the test results, we chose heart tissue corresponding to the ventricular site, and the sampling location and size between groups were largely the same. Since the adult offspring of the control and ART groups appeared to have the normal phenotype and there were no obvious individual differences between the same-sex animals, we set up only three biological replicates from different litters for sequencing. Unexpectedly, the sequencing quality of one spleen sample in the ART group was suboptimal, which maybe resulted from the testing system or human factors. Considering the deviation caused by the batch effect, we did not send additional samples for retesting. The small number of biological replicates may affect the statistical values to a certain extent, which is a limitation of this study. RNA sequencing (RNA-seq) showed a small amount of abnormal gene expression in the spleen of the ART offspring, while there were a large number of abnormally expressed genes in the heart, which to some extent explains why ART increases the risk of cardiac dysfunction and disease (43, 44). It has been reported that IVF and embryo culture altered the gene expression in pancreatic islets and insulin-sensitive tissues (liver, skeletal muscle, and adipose tissue), involving multiple classical signal pathways (8). Through enrichment analysis, we found that the biological processes with the most significant enrichment of cardiac DEGs were related to RNA transcriptional synthesis, which suggests that the effects of ART on the cardiac function of offspring may be multifaceted and profound. “Cardiovascular system development” was also markedly enriched, and an interaction analysis found some core genes including *Mapk14*, which encodes the protein kinase MAPK14 (P38α) that plays an important role in initiating cardiovascular and other diseases. Consequently, its inhibitors can be used to treat diseases (45). However, it has been reported that the inhibition of p38 and the JNK pathways can cause myocardial hypertrophy (46). ROCK1, another protein kinase, affects cardiovascular physiological and pathological conditions, which means that a ROCK inhibitor can play a therapeutic or protective role (47). Under high glucose conditions *in vitro* or *in vivo*, ROCK1 can mediate cardiomyocyte apoptosis through P53/NOXA, which may cause ventricular wall thinning (48). Although myocardial contraction and cardiac signaling were not among the significant enrichment terms, several key DEGs were involved in these pathways. A previous study also analyzed the effect of IVF-ET on the cardiac transcriptome of adult offspring (49). Different from our research, it did not include oocyte vitrification and thawing procedures; the culture medium was suboptimal Whitten’s medium, in which the IVF embryos were cultured until blastocyst stage; the gene expression changes were detected in the hearts of elderly male mice. Nonetheless, it also identified more than 1000 affected genes containing the 16 genes that were the most significantly different (FC>2), 2 of which (i.e. Ptgs2 and Ccl7) also appeared to be differential genes in our study and require further attention. Although the differentially expressed genes and the enrichment analysis results may be influenced by different experimental designs, both are involved in how the circulatory system develops and works. Differential genes in the spleen are mainly enriched in immunity and anti-infection. Differential expressed transcription factor pairs include FOS and JUN, both of which are constituent subunits of transcription factor AP-1. Fos-deficient mice are susceptible to *Salmonella typhimurium* infection (50). AP-1 is an important regulator of some immune disorders and cancer and is a marker of potential disease occurrence and development (51, 52).

In recent years, the potential effects of ART on epigenetic modifications in early embryos and offspring have received extensive attention. DNA methylation and histone modification are closely related to gene expression changes in physiological and pathological processes (53, 54). Combined with relevant databases, we specifically analyzed the effect of ART on the expression of epigenetic modifiers and imprinted genes. The results showed the abnormal expression of several epigenetic modifiers in the organs of the ART offspring. The heart contained SMARCA5, ARID4B, KDM2A, SETD1B, and other key nodes, while the spleen contained KDM6A, KDM5D, and UTY, which interact with each other. All the above factors were related to histone modification or chromatin remodeling. There were no significant abnormalities in DNA methylation-related factors. Therefore, the aberrantly expressed genes in ART offspring may be significantly regulated by histone modifications and chromatin remodeling factors. However, several studies have shown that ART procedures affect DNA methylation in early embryos and offspring tissues. The addition of melatonin in embryo culture medium can prevent vascular dysfunction and artistic hypertension in ART mice, which may be achieved by changing the methylation and expression of the promoter of the endodermal nitric oxide synthase (eNOS) gene (17). The methylation disorder of genes related to liver development in ART embryos may cause abnormal lipid metabolism in livers and increase the risk of fatty liver (55). There were methylation abnormalities in genes related to liver development in embryos after PGT, some of which are related to glucose homeostasis and insulin response, increasing the risk of liver-derived insulin resistance in the offspring (56). In fact, there is interplay between DNA methylation and histone modification (57), so the change of DNA methylation may also be regulated by histone modification. The effect of ART on the histone modification of offspring needs to be further studied.

Studies have shown that the establishment, maintenance, and expression of imprinted genes not only require specific DNA methylation patterns, but also correct histone modifications (58). It was found that the expression of imprinted genes in the spleen was not affected. However, there were four imprinted genes in the heart with abnormally low expression, of which *Dhcr7* is the paternal imprinted gene and the others are maternal, such as *Igf2* and *Mest*. The expression of the imprinted gene Slc38a4 in the adipose tissue of female adult offspring decreased after embryo culture *in vitro* (37).ART reduced the fetal growth associated with the downregulation of maternal imprinted genes that should be enhanced in fetal growth of mice (59). The IGF2 gene is related to cardiac vascular regeneration in type 2 diabetes cardiomyopathy (60). Maternal malnutrition in the third trimester of pregnancy increased IGF2 signaling molecules and collagen deposition in the right ventricle of fetal sheep, which may contribute to myocardial remodeling of the right ventricle, thus having a negative effect on heart health in later years (61). Myocardial morphological studies showed that the trabecular meshwork formation pattern in mice with the *Mest* gene deletion was slightly changed, similar to human cardiac myopathy ventricular noncompaction (62). Our further study showed that the DNA methylation levels of *Igf2* and *Mest* ICRs were abnormally elevated in the heart of the ART offspring. The methylation levels of the imprinted genes *H19/Igf2* and *Peg3* were abnormal in ART children aged 7 to 8 (63). When the screening standard of the DEGs was set to FC > 2, the expression of DNA demethylase TET3 in the heart of ART offspring significantly decreased (FDR < 0.01). Therefore, DNA methylation- and histone modification-related factors may jointly participate in regulating the expression and methylation of imprinted genes in ART offspring.

In conclusion, this study revealed ART-affected gene expression in certain key pathways in the heart and spleen of the offspring, including epigenetic modifiers and developmentally important imprinted genes. The above changes may have adverse consequences for the offspring. This study provided experimental data and act as a reference for evaluating the safety of human ART and further optimizing the ART system.

## Data availability statement

The datasets presented in this study can be found in the GEO repository (https://www.ncbi.nlm.nih.gov/geo/), accession number GSE211597.

## Ethics statement

The animal study was reviewed and approved by The Life Science Ethics Review Committee of Zhengzhou University.

## Author contributions

LM and CZ designed the study. HC and LZ performed the experiment, prepared samples for sequencing, and wrote the manuscript. FY collected samples and performed English editing and data analysis. CC performed data processing and analysis. YL collected samples. QZ and LL edited and revised the manuscript. All authors contributed to the article and approved the submitted version.
