# Supplementary material for: Effects of assisted reproductive technology on gene expression in heart and spleen tissues of adult offspring mouse
Source: Front Endocrinol (Lausanne). 2023 Mar 30;14:1035161. doi: 10.3389/fendo.2023.1035161 (PMC10098333; doi:10.3389/fendo.2023.1035161)
Supplement: Supplementary file 2 [file Table_2.docx]

**Table S2.** Primer sequences for real-time fluorescent quantitative PCR (qPCR).

| Genes | Primer Sequences | Annealing Temperature | Product Lengths |
| --- | --- | --- | --- |
| *GAPDH*  *IGF2*  *MEST*  *KDM5D*  *KDM2A*  *MAPK14*  *ROCK1*  *FOS*  *JUN* | F: AATGGTGAAGGTCGGTGTGA  R: TGATGGGCTTCCCGTTGATG  GGACCGCGGCTTCTACTTC  GTATCTGGGGAAGTCGTCCG  CCTGTGATCCGCAATCCTGC  CCTCTGACCAAGTGAATCCCC  GGCAACCTCCTTTTGCTGTAGA  ACGGTCCTTGCAGATTGCTTC  AGTAGCCGAGTGGTCTTGCT  TCCAGGGATGCCTTCCTCTT  AAGACTCGTTGGAACCCCAG  GGGTCGTGGTACTGAGCAAA  AACGCTCCGAGACACTGTAG  CTTTCCTGCAAGCTTTTATCCA  TGTTCCTGGCAATAGCGTGT  TCAGACCACCTCGACAATGC  GCACATCACCACTACACCGA  GGGAAGCGTGTTCTGGCTAT | 58℃  58℃  58℃  58℃  58℃  58℃  58℃  58℃  58℃ | 218bp  187bp  127bp  242bp  248bp  234bp  243bp  170bp  127bp |
