# Supplementary material for: Effects of assisted reproductive technology on gene expression in heart and spleen tissues of adult offspring mouse
Source: Front Endocrinol (Lausanne). 2023 Mar 30;14:1035161. doi: 10.3389/fendo.2023.1035161 (PMC10098333; doi:10.3389/fendo.2023.1035161)
Supplement: Supplementary file 3 [file Table_3.docx]

**Table S3.** Nested PCR with two primer pairs for Bisulfite Sequencing PCR (BSP).

| Genes | Primer Sequences | Annealing Temperature | Product Lengths |
| --- | --- | --- | --- |
| *Mest* outer  *Mest* inner  *IGF2* outer  *IGF2* inner | OF: GATTTGTTATATAAAAGGTTAATGAG  OR: TCATTAAAAACACAAACCTCCTTTAC  IF: TTTTAGATTTTGAGGGTTTTAGGTTG  IR: AATCCCTTAAAAATCATCTTTCACAC  GAGTATTTAGGAGGTATAAGAATT  ATCAAAAACTAACATAAACCCCT  GTAAGGAGATTATGTTTATTTTTGG  CTAACCTCATAAAACCCATAACTAT | 50℃  55℃  50℃  50℃ | 690bp  564bp  474bp  427bp |
