# Supplementary material for: Effects of assisted reproductive technology on gene expression in heart and spleen tissues of adult offspring mouse
Source: Front Endocrinol (Lausanne). 2023 Mar 30;14:1035161. doi: 10.3389/fendo.2023.1035161 (PMC10098333; doi:10.3389/fendo.2023.1035161)
Supplement: Supplementary file 4 [file Table_4.docx]

**Table S4.** Quantity and quality of sequenced data.

| Sample | Raw Data(bp) | Clean Data(bp) | Q20(%) | Q30(%) |
| --- | --- | --- | --- | --- |
| HC1  HC2  HC3  HT1  HT2  HT3  SC1  SC2  SC3  ST1  ST2 | 7007096100  6970391700  7169493000  7350975900  7167810900  6749210400  7732266300  6354150000  6312428100  5899026600  6935621400 | 6838327696  6835443572  7009640302  7203599223  7030366757  6614975668  7604041242  6094850432  6187544201  5677452616  6741186450 | 6708585344 (98.10%)  6706862786 (98.12%)  6880047559 (98.15%)  7056818143 (97.96%)  6889499004 (98.00%)  6485756312 (98.05%)  7437165545 (97.81%)  5962904854 (97.84%)  6067093763 (98.05%)  5563506885 (97.99%)  6610983269 (98.07%) | 6459632966 (94.46%)  6457234547 (94.47%)  6627931822 (94.55%)  6770703097 (93.99%)  6617441312 (94.13%)  6232691283 (94.22%)  7122013747 (93.66%)  5725304461 (93.94%)  5844536708 (94.46%)  5357662692 (94.37%)  6370243844 (94.50%) |
