# Supplementary material for: Effects of assisted reproductive technology on gene expression in heart and spleen tissues of adult offspring mouse
Source: Front Endocrinol (Lausanne). 2023 Mar 30;14:1035161. doi: 10.3389/fendo.2023.1035161 (PMC10098333; doi:10.3389/fendo.2023.1035161)
Supplement: Supplementary file 5 [file Table_5.docx]

**Table S5.** RNA-seq read filtration and genome mapping.

| Sample | Raw_Read | Clean_Read(%) | Mapped_Reads(%) | Unique_Mapped(%) | Covered_Genes(%) |
| --- | --- | --- | --- | --- | --- |
| HC1 | 46713974 | 45903710(98.27%) | 44722838(97.77%) | 38675711(84.55%) | 15620(70.05%) |
| HC2 | 46469278 | 45827768(98.62%) | 44612238(97.72%) | 38218729(83.72%) | 15669(70.27%) |
| HC3 | 47796620 | 47062410(98.46%) | 45855544(97.75%) | 39700742(84.63%) | 15665(70.25%) |
| HT1 | 49006506 | 48310352(98.58%) | 46813324(97.66%) | 34945301(72.90%) | 15612(70.02%) |
| HT2 | 47785406 | 47149300(98.67%) | 45722210(97.40%) | 36625655(78.02%) | 15964(71.59%) |
| HT3 | 44994736 | 44333474(98.53%) | 43119564(97.64%) | 33945096(76.86%) | 15725(70.52%) |
| SC1 | 51548442 | 50960696(98.86%) | 49099917(96.55%) | 44794087(88.09%) | 16520(74.09%) |
| SC2 | 42361000 | 40840948(96.41%) | 39045914(95.80%) | 35822113(87.89%) | 16430(73.68%) |
| SC3 | 42082854 | 41508820(98.64%) | 39868406(96.26%) | 36510789(88.15%) | 16464(73.84%) |
| ST1 | 39326844 | 38168752(97.06%) | 36308772(95.75%) | 33452173(88.21%) | 16328(73.23%) |
| ST2 | 46237476 | 45152336(97.65%) | 43231198(96.34%) | 39085786(87.10%) | 16380(73.46%) |
